# *AutoPPI*: An Ensemble of Deep Autoencoders for Protein–Protein Interaction Prediction

**DOI:** 10.3390/e23060643

**Published:** 2021-05-21

**Authors:** Gabriela Czibula, Alexandra-Ioana Albu, Maria Iuliana Bocicor, Camelia Chira

**Affiliations:** Department of Computer Science, Babeş-Bolyai University, 400084 Cluj-Napoca, Romania; gabriela.czibula@ubbcluj.ro (G.C.); alexandra.albu@ubbcluj.ro (A.-I.A.); maria.bocicor@ubbcluj.ro (M.I.B.)

**Keywords:** deep learning, autoencoders, protein–protein interaction

## Abstract

Proteins are essential molecules, that must correctly perform their roles for the good health of living organisms. The majority of proteins operate in complexes and the way they interact has pivotal influence on the proper functioning of such organisms. In this study we address the problem of protein–protein interaction and we propose and investigate a method based on the use of an ensemble of autoencoders. Our approach, entitled AutoPPI, adopts a strategy based on two autoencoders, one for each type of interactions (positive and negative) and we advance three types of neural network architectures for the autoencoders. Experiments were performed on several data sets comprising proteins from four different species. The results indicate good performances of our proposed model, with accuracy and AUC values of over 0.97 in all cases. The best performing model relies on a Siamese architecture in both the encoder and the decoder, which advantageously captures common features in protein pairs. Comparisons with other machine learning techniques applied for the same problem prove that AutoPPI outperforms most of its contenders, for the considered data sets.

## 1. Introduction

All molecular interactions in a cell have been termed the interactome. Most such interactions involve proteins, which can bind to other proteins or to small molecules. Among these, the interactome of protein–protein interactions (PPI) is of particular significance, as more than 80% of proteins perform their roles not individually, but in complexes [1] and thus this interactome can unveil interactions that are connected to diseases and also which genes are involved [2]. In addition, the interactome can assist in identifying functions of unknown proteins, considering that proteins that interact are often times involved in similar cellular processes [3]. Thus, by determining all interactions of a new protein, one can infer its function, assuming that the interacting proteins are already accounted for. Going one step further, interaction information can be instrumental in the field of drug design, as knowledge about certain proteins’ interactions and binding sites can be used to architect drugs that target those specific proteins.

Methods for the identification of PPI can be classified into three categories: in vivo, in vitro and in silico [1]. The first one involves methods performed on whole organisms (simpler organisms, e.g., yeast), the second includes chemical and physical mechanisms performed in a controlled environment (affinity chromatography, coimmunoprecipitation, protein microarrays, X-ray crystallography, nuclear magnetic resonance (NMR) spectroscopy) [1], while the third refers to computational methods, experiments and simulations. In vivo and in vitro methods offer reliable results, however they also have disadvantages such as false positives or negatives, lack of possible PPI, the necessity for extra-validation of obtained results or high costs. As a consequence, in silico methods were developed both to complement the first two more traditional techniques, but also to act independently for the identification of PPI. Computational techniques include approaches starting from biological sources such as protein sequences, structures, co-evolution of proteins, phylogenetic profiles, protein or gene ontologies, gene fusion, gene annotations or various combinations of these [4,5,6,7,8,9,10]. As opposed to three-dimensional structures, primary sequences (the chain of amino acids) are known for having a substantial number of proteins, thus the sequence data constitutes a simple and available start source.

Machine learning methods are among the most popular choices for addressing this problem. Many researchers have pointed their attention in this direction and there are numerous studies approaching PPI that work with protein sequences. Earlier research focuses on “traditional” learning techniques such as Bayesian networks [11], support vector machines [12], random forests [13] or k-nearest neighbour [14]. Some recent approaches also use such techniques in different combinations; for instance, in [15] the authors propose a two-level stacked solution that integrates five classifiers. However, recent advances in the field of deep learning and the versatility and generality of such methods make them more than suitable candidates for proposing potential solutions to the problem at hand.

We propose and investigate a method based on the use of autoencoders with the aim to tackle the underlying classification problem in relation to PPI. The proposed approach, called AutoPPI, consists of data collection, feature selection, training of two autoencoders (one focusing on the class of proteins that interact and the other on proteins that do not interact) and performance evaluation. Three neural network architectures are proposed for the autoencoders and experiments are performed for several PPI data sets. The obtained results support a competitive performance of the new AutoPPI classification model showing that autoencoders can capture relationships between proteins relevant for their interactions and can successfully be used by themselves for PPI prediction.

The main innovation our work brings, compared to the methods previously proposed, resides in the manner in which autoencoders are involved in the process of PPI prediction. While heretofore the main task of autoencoders was feature extraction, the actual classification being performed by an additional classifier (as presented in Section 2), in our approach these self supervised techniques are the main actors. We train an autoencoder for each class in the input data (interacting and non-intersecting proteins) and further employ these to compute, for each pair of proteins in the test set, a probability that will indicate whether they are prone to interact or not. To the best of our knowledge, autoencoders have not been used solo for the PPI task, so far.

## 2. Literature Review

Protein sequence information is easily deduced from DNA using the genetic code. For most discovered proteins this type of information is known: the most comprehensive non-redundant protein sequence database currently contains 174 million sequences [16] and is doubling in size every 28 months [17]. However, determining protein structure is a complex process and thus there are considerably fewer structures available—approximately 175,000 structures in the Protein Data Bank [18]. Approaches for determining PPI that start from sequence information not only have more data to work with, but can also be considered more general, as they do not need additional information such as structure, functions or annotations. Considering that in this research we investigate deep autoencoders and employ protein sequences, we direct our focus towards deep learning methods for PPI, which also start from sequence data. Note that this is relevant not only with regard to the data sets we use to experimentally evaluate our proposed methods, but also for the type of encoding that must transform the input data into numerical representation for the learning algorithm.

Chen et al. [19] approach multi-class PPI via a framework that includes a siamese deep residual recurrent convolutional neural network (to capture latent features of sequence pairs) and a data processing component, and which preserves contextualised and sequential information found in proteins. Protein sequences are encoded using pre-trained Skip-Gram embeddings which capture amino acids co-occurence similarities and one-hot encodings of hydrophobicity and electrotaticity classes [20]. Several data sets of different sizes are employed for various types of prediction tasks (binary, multi-class interaction and binding affinity estimation).

To leverage the strengths of several techniques and to achieve a more comprehensive procedure, Li et al. [21] introduce a a deep ensemble learning method. The method uses a combination of encodings for proteins (local descriptors, auto covariance, conjoint triads, pseudo amino acid composition) and an ensemble model including several modules such as: input, convolution, attention mechanism (which uses the multi-attention mechanism to capture essential features), deep neural network and, lastly, an integration module. The evaluation is performed on five data sets and the obtained prediction performances demonstrate the strengths of the proposed method.

Autoencoders in particular have not been widely adopted to tackle PPI, but there are some recent papers that propose solutions based on this type self-supervised learning methods. Two works by Wang et al. [22,23] use autoencoders in the process of predicting PPI. The main predictor is a probabilistic SVM, whose input is provided by the autoencoders. In both approaches numeric matrices extracted from protein sequences are brought into play (in one case, the position specific scoring matrix and in the other the position weight matrix). From these, the authors extract another level of information via Zernike moments and Legendre moments, respectively, which are further refined via a stacked autoencoder. Lastly, the obtained extracted features are fed to the SVM-based classifier. In both cases the method was tested via several protein data sets and the resulting accuracies are high.

A stacked autoencoder as for PPI classification is proposed by Sun et al. [7]. The input is provided by applying either autocovariance or conjoint triads and the autoencoder latent representation is linked to a softmax classifier. After the training phase, the authors notice that models containing just one hidden layer were sufficient for relatively high accuracy. Several test sets from different species have been experimented on and the results suggest that the method obtains superior accuracies, compared to other methods.

Sharma and Singh [24] propose the use of autoencoders in conjunction with Light Gradient Boosting Machine (LightGBM) for PPI prediction. They combine protein sequence features obtained by conjoint triads and a multi-descriptor called composition-transition-distribution and employ the autoencoder to reduce the dimentionality of the vectors fed to LightGBM. Experiments were performed on six PPI data sets, as well as three PPI networks and the proposed model obtained very good performances.

Variational autoencoders have also proved their value in PPI prediction, as demonstrated by Yang et al. [25]. However, as opposed to the previously mentioned research, this work is different in that it also needs structural information of PPI networks. The main classifier in this case is a feedforward artificial neural network which receives as input protein embeddings created by a signed variational graph autoencoder (S-VGAE). Similar to many other research before, the proteins are first encoded using the conjoint triads method and further the S-VGAE learns embeddings for proteins based on their sequences and graph information (such as position, neighbouring nodes). The obtained results show that the proposed approach outperforms other methods in the literature, yet it must be noted that this method also requires additional input information.

Autoencoders are neural networks which learn to reconstruct the input data, typically by mapping the input to a compressed representation. From this perspective, autoencoders learn nonlinear embeddings for the inputs, which are able to capture the essential characteristics of the input data [26]. Paired data instances are usually modeled by siamese architectures, which are networks designed to learn shared weights between the two instances in a pair. Siamese neural networks have been successfully used for one-shot classification of images [27] and sentence matching [28]. Siamese versions of autoencoders have been studied for tackling various problems, however, they differ from our proposed architectures. A siamese autoencoder composed of a shared encoder and two separate decoders—one for each component in the pair—has been introduced by Utkin et al. [29] for detecting anomalies in multi-robot systems. Variational siamese networks were proposed by Deudon [28] for effectively learning semantic similarities between questions. In their work, variational autoencoders are used to map questions to their reformulations. Afterwards, the pre-trained encoder is used as the siamese component of a neural network trained to predict semantic similarity of two questions.

## 3. Methodology

In this section we are introducing a binary supervised classifier AutoPPI for predicting if two proteins interact or not. The proposed classifier is composed of two *autoencoders* (AEs) used for encoding relationships between both the class of proteins that interact and the class of proteins that do not interact.

We decided to use autoencoders in designing AutoPPI due to their ability to self-supervisedly learn, through their latent space representation, features that are relevant for distinguishing between pairs of proteins that interact or not.

### 3.1. Theoretical Model

The PPI problem may be formalized as a binary classification one. Let us consider that we are given two classes $C_{+}$ (the *positive* class) and $C_{-}$ (the *negative* class), where by $C_{+}$ we denote the class consisting of pairs of proteins that interact and $C_{-}$ is composed by all pairs of proteins that do not interact. The PPI problem formalized as a binary classification problem consists of deciding if a given pair of proteins ($p_{1}$, $p_{2}$) belongs to the *positive* class or to the *negative* class. Let us denote, in the following, by C the set of all pairs of proteins, i.e., $C=C_{+}\cup C_{-}$.

From a machine learning perspective, the PPI classification problem may be formalized as learning to approximate two target functions $pr_{+}:C\to \left[0,1\right]$ and and $pr_{-}:C\to \left[0,1\right]$ expressing the probability that a certain pair of proteins belongs to either the “+” or the “−” class, i.e., $pr_{+}\left(p\right)+pr_{-}\left(p\right)=1,\;\;\forall p\in C$.

In a supervised learning scenario, the aim is to train a binary classifier on pairs of proteins belonging to both $C_{+}$ and $C_{-}$ with the goal of predicting if a pair of proteins unseen during training belongs to $C_{+}$ (i.e., the proteins interact) or to $C_{-}$ (i.e., the proteins do not interact).

Our AutoPPI classifer uses two autoencoders $A_{+}$ and $A_{-}$, the first one being trained to learn relationships between the proteins that interact while the second one is trained to recognize pairs of proteins that do not interact. The aim is to train the classifier to predict if a certain pair of proteins does or does not interact, the prediction being based on the similarity degree of the given pair of proteins against all other pairs of proteins that are encoded into the autoencoders $A_{+}$ and $A_{-}$. The autoencoders are used, through the representation of their hidden (encoded) state, for learning relevant characteristics and discriminating between pairs of proteins that interact and pairs of proteins that do not.

**Figure 1:**
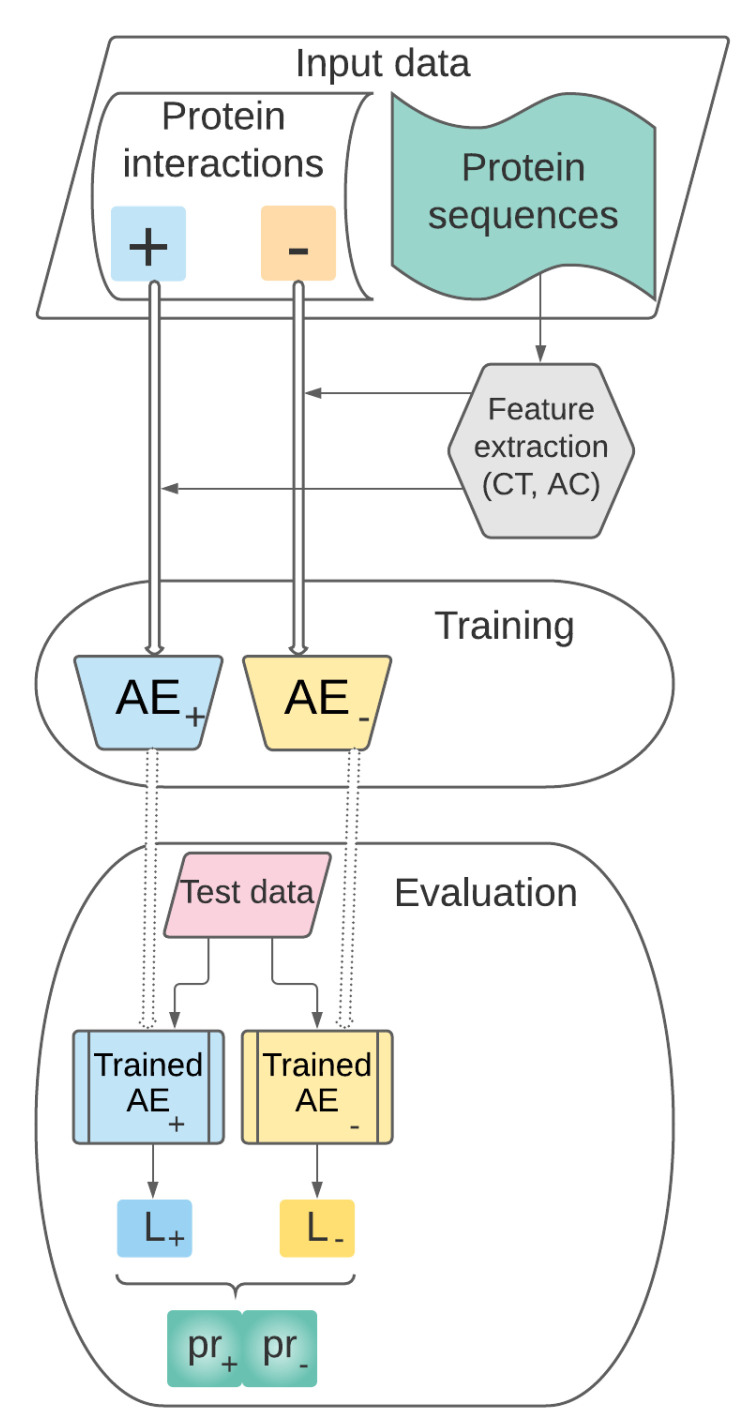
Overview of our approach: AutoPPI.

As depicted in Figure 1, the main stages of AutoPPI are as follows:

1. **Data collection, representation and preprocessing**. This stage includes the following steps:
  i. Collection of data sets which will be used in further training AutoPPI (i.e., the pairs of proteins from C);
  ii. Selection of the set of features relevant for representing the pairs of protein sequences in a vector space model.
2. **Training**. The set of preprocessed vectors characterizing pairs of proteins prepared at the previous stage will be used for training the AEs and for building the supervised learning model AutoPPI.
3. **Performance evaluation**. This stage refers to the performance evaluation of our predictive model AutoPPI previously trained. AutoPPI will be tested on pairs of proteins unseen during the training stage and its performance will be assessed through relevant evaluation metrics.

The following sections will detail the stages of our approach.

### 3.2. Data Collection, Representation and Preprocessing

Let us consider that a protein is encoded by a set $F=(F_{1},F_{2},\cdots F_{k})$ of relevant features. Thus, a protein *p* will be represented as a numerical high-dimensional vector $p=(p_{1},p_{2},\cdots ,p_{k})$, where $p_{i}$ represents the value of feature $F_{i}$ obtained for protein *p*. A pair of proteins $\left(p,p^{\prime }\right)$ will be, subsequently, visualized as a data point in $R^{2\cdot k}$, i.e., a 2·k dimensional vector obtained by concatenating the *k*-dimensional representation of proteins *p* and $p^{\prime }$. More specifically, if $p=(p_{1},p_{2},\cdots ,p_{k})$ and $p^{\prime }=\left(p_{1}^{\prime },p_{2}^{\prime },\cdots ,p_{k}^{\prime }\right)$ then the pair $\left(p,p^{\prime }\right)$ is represented as the vector $\left(p_{1},p_{2},\cdots ,p_{k},p_{1}^{\prime },p_{2}^{\prime },\cdots ,p_{k}^{\prime }\right)$.

For selecting the most appropriate feature-based representation for proteins, we started from two representations extensively used in protein–protein interaction prediction tasks: Conjoint Triad (CT) descriptors [7,20,30] and Autocovariance (AC) descriptors [7,12,15,30].

1. **CT features** [20] can be used to obtain fixed-length representations for protein sequences by grouping amino acids into seven classes based on their physico–chemical properties. Then, a sliding window of size 3 is passed through the protein sequence and the frequencies of possible triples of amino acid classes are computed. Thus, for a protein, a vector of size 7×7×7=343 is built. Since longer protein sequences are more likely to have higher frequency values than shorter sequences, the final values of the CT descriptors are represented by the normalized frequencies. The CT descriptors were obtained using the *iFeature* library [31].
2. **AC features** are another type of descriptors which characterize variable-length protein sequences using vectors of fixed size [12]. Unlike CT features which take into account only groups of three consecutive amino acids, AC descriptors are able to capture long-term dependencies in a protein sequence, through defining a lag variable and computing correlations between amino acids situated in the sequence at at most lag positions apart. Thus, for *m* properties and a distance lag, a vector of size m×lag is obtained. We computed the AC features using the group of 14 amino acid properties provided by Chen et al. [15]. These properties are hydrophobicity computed using two different scales, hydrophilicity, net charge index of side chains, two scales of polarity, polarizability, solvent-accessible surface area, volume of side chains, flexibility, accessibility, exposed surface, turns scale and antegenic propensity [15]. Since the value of lag needs to be smaller than the sequence length, we selected different values, according to each tested data set (details are provided in Section 4).
  In the computation of both descriptors we used the default procedure of *iFeature* which removes non-standard amino-acids from the protein sequences.

In this study, we considered a combined feature-based representation for the proteins, obtained by concatenating the AC and CT features computed for a protein sequence, in order to leverage the information captured by both types of features. The chosen method of representation uniformizes proteins, irrespective of their sequence length.

### 3.3. Training

As previously described, an autoencoder is trained for each class $C_{i}$ (i∈{+,−}) and is aimed to encode relationships between the features characterizing proteins that do or do not interact. Let us denote by $AE_{+}$ and $AE_{-}$ these autoencoders: $AE_{+}$ will be trained on the data set $C_{+}$, while $AE_{-}$ will be trained on the data set $C_{-}$. A training example consists of the 2·k-dimensional vectorial representation (see Section 3.2) for the pair of proteins labeled with the class to which the pair of proteins belongs (i.e., “+” or “−”). During the autoencoders’ training, a loss function that penalizes the difference between the output generated by the autoencoder and the provided input is used such that the autoencoders will learn to encode pertinent relationships between pairs of proteins.

For training AutoPPI, for each i∈{+,−}, we will use the majority (either 80% or 90%, according to the tested data set) of the data set $C_{i}$ for training $AE_{i}$ and validation and the remaining data (20%, 10%) from $C_{i}$ will be subsequently used for testing. From the training and validation portion of the data, we randomly selected a proportion of 10% for model validation.

#### 3.3.1. Proposed AE Architectures

Three architectures are proposed for the autoencoders $AE_{+}$ and $AE_{-}$ and these are detailed in the following sections.

**Joint–Joint architecture**

**Figure 2:**
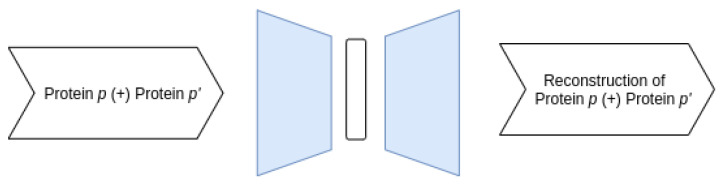
Overview of the Joint–Joint architecture.

Our first architecture receives as input data the representation describing the pairs of proteins and reconstructs the concatenated features corresponding to the protein pairs. A schematic representation of the architecture is depicted in Figure 2.

**Siamese–Joint architecture**

Guided by the success of siamese neural network architectures in modeling pair data [19,32,33,34], we designed two autoencoder architectures aimed at better capturing common features in the protein pairs. Thus, instead of directly combining the protein features in the input space, these architectures have a shared encoder structure which compresses the two proteins in a pair in two encodings, thus being able to capture patterns present in both proteins from a pair.

**Figure 3:**
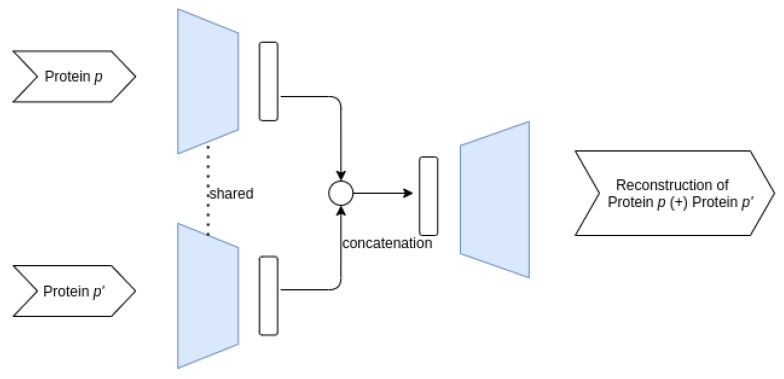
Overview of the Siamese–Joint architecture.

The first such architecture, further referred to as Siamese–Joint, has a shared structure only for the encoder, while aiming to reconstruct the concatenated features of the two proteins. This architecture is presented in Figure 3. The shared encoder compresses the two proteins *p* and $p^{\prime }$ into the latent space representations *z* and $z^{\prime }$, which are subsequently concatenated and used to reconstruct the pair ($p,p^{\prime }$).

**Siamese–Siamese architecture**

**Figure 4:**
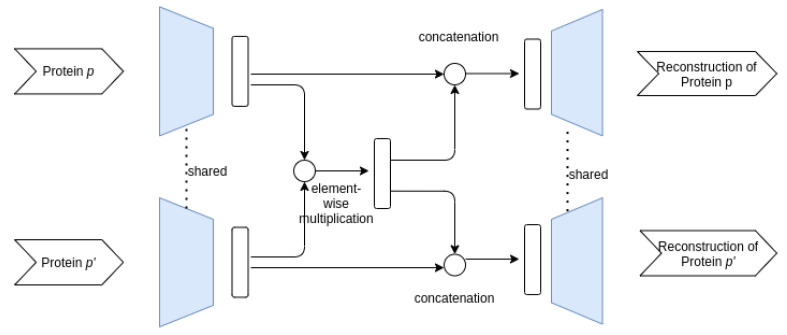
Overview of the Siamese–Siamese architecture.

The last investigated architecture has a siamese structure in both the encoder and decoder, the weights being shared between the two proteins in a pair. In order to encode information about both proteins in a pair, we obtain a reconstruction for each protein by also using the other protein in the pair. This is achieved by combining the encodings of the two proteins into a common encoding that is used for reconstructing the original proteins. Figure 4 depicts the autoencoder architecture. The encodings *z* and $z^{\prime }$ for *p* and $p^{\prime }$ respectively are multiplicated element-wise, yielding a common representation $\hat{z}$. Then, the reconstruction of *p* is obtained from the concatenation of *z* and $\hat{z}$ and the reconstruction of $p^{\prime }$ is obtained from the concatenation of $z^{\prime }$ and $\hat{z}$. The last concatenation step is performed in order to obtain different encodings for the two proteins in the pair to be passed through the shared decoder.

The neural network architectures employed in our study are formed of fully connected layers. For all of the three above mentioned autoencoder architectures, the encoder was formed of two layers of 600 neurons linked to a bottleneck layer of 300 neurons, followed by a symmetric decoder. The SELU [35] activation function was used. The autoencoders were trained by minimizing the mean squared error loss using the Adam optimizer with an initial learning rate of 0.0005 and batch size of 64 for 2000 epochs. The learning rate was reduced by a factor of 2 when reaching a sequence of 5 epochs during which the validation loss had not improved, up to a minimum value of $10^{-5}$. The models were implemented using the TensorFlow library [36].

## 3.4. Performance Evaluation

This section introduces the methodology applied for evaluating the performance of AutoPPI model after it was trained as shown in Section 3.3. We start by explaining in Section 3.4.1 how the classification of a new pair of proteins will be provided by the trained AutoPPI. Then, the evaluation metrics and the testing methodology will be introduced.

### 3.4.1. Classification Using AutoPPI

At the testing stage of the previously trained AutoPPI classifier, a new pair of proteins $\left(p,p^{\prime }\right)$ has to be classified as belonging to the “+” or “−” class. As shown in Section 3.2, the pair $\left(p,p^{\prime }\right)$ is represented as an 2·k-dimensional numerical vector consisting of the concatenated list of relevant features characterizing the proteins *p* and $p^{\prime }$. We decide if two proteins *p* and $p^{\prime }$ are likely to interact if the loss of autoencoder $AE_{+}$ computed for the pair (*p*, $p^{\prime }$) is lower than the loss of autoencoder $AE_{-}$ computed for the same pair. This means, intuitively, that the pair (*p*, $p^{\prime }$) is likely to be more similar to the information encoded by $AE_{+}$ than to the one encoded by $AE_{-}$. The underlying idea is the fact that an autoencoder is known to be able to reconstruct data selected from the same distribution as the data it was trained on. Moreover, the autoencoder is unable to recreate, using its learned hidden representation, an input instance dissimilar to the training data. The classification of AutoPPI is based on computing two probabilities: $pr_{+}\left(p,p^{\prime }\right)$ representing the probability that the proteins *p* and $p^{\prime }$ interact and $pr_{-}\left(p,p^{\prime }\right)$ representing the probability that the proteins *p* and $p^{\prime }$ do not interact.

Let us denote by $L_{-}\left(p,p^{\prime }\right)$ the loss value computed for the pair (*p*, $p^{\prime }$) by the autoencoder $AE_{-}$ and by $L_{+}\left(p,p^{\prime }\right)$ the loss value computed for the pair (*p*, $p^{\prime }$) by the autoencoder $AE_{+}$.

The probabilities $pr_{-}\left(p,p^{\prime }\right)$ and $pr_{+}\left(p,p^{\prime }\right)$ are computed as given in Formulas (1) and (2).

(1) $$pr_{-}\left(p,p^{\prime }\right)=0.5+\frac{L_{+}\left(p,p^{\prime }\right)-L_{-}\left(p,p^{\prime }\right)}{2\cdot (L_{+}\left(p,p^{\prime }\right)+L_{-}\left(p,p^{\prime }\right))}$$

(2) $$pr_{+}\left(p,p^{\prime }\right)=1-pr_{-}\left(p,p^{\prime }\right).$$

From Formula (1) we note that $0\leq pr_{-}\left(p,p^{\prime }\right)\leq 1$ and that if $L_{-}\left(p,p^{\prime }\right)\leq L_{+}\left(p,p^{\prime }\right)$, then $pr_{-}\left(p,p^{\prime }\right)\leq 0.5$, meaning that the pair (*p*, $p^{\prime }$) is classified by AutoPPI as being *negative* (i.e., there is no interaction between proteins *p* and $p^{\prime }$ ). Moreover, $pr_{-}\left(p,p^{\prime }\right)=1$ if $L_{-}\left(p,p^{\prime }\right)=0$.

### 3.4.2. Testing

After it was trained, AutoPPI is tested on the remainder of each data set $C_{+}$ and $C_{-}$ which was not used for training or validation. For each testing set, the confusion matrix is computed for the binary classification task and it consists of the following values: TP (*true positives*), FP (*false positives*), TN (*true negatives*) and FN (*false negatives*). The classification of a certain pair of proteins (*p*, $p^{\prime }$) is decided as described in Section 3.4.1.

For measuring the performance of AutoPPI on a certain testing data set, the following evaluation measures are reported based on the *confusion matrix* values (TP, TN, FP and FN). Based on the values from the confusion matrix, the following evaluation measures are computed [37]:

- **precision**, $Prec=\frac{TP}{TP+FP}$;
- **recall** or *sensitivity*, $Recall=\frac{TP}{TP+FN}$;
- **specificity** or *true negative rate*, $Spec=\frac{TN}{TN+FP}$;
- **F1-score** or *F-score*, $F_{1}=2\cdot \frac{precision\cdot recall}{precision+recall}$;
- **Area Under the ROC Curve** (AUC), $AUC=\frac{Spec+Recall}{2}$.

Due to the randomness involved in the selecting the training/validation/testing data sets, a *k-fold cross-validation* testing methodology is applied by repeating the testing *k* times. The values for the evaluation measures are then averaged over the *k* runs and a 95% *confidence interval* (CI) [38] of the average value is computed. We used the same number of folds used in the literature for those data sets.

## 4. Experimental Results

This section starts by describing in Section 4.1 the data sets used for evaluating the performance of the AutoPPI classifier introduced in Section 3. The experimental results obtained from the considered case studies are then presented in Section 4.2.

### 4.1. Data Sets

The first data set used in our experiments is Pan’s human protein–protein interactions data set [39], which contains positive samples collected from the **HPRD-2007** database. The negative interactions were obtained by selecting pairs of proteins with different sub-cellular localizations [7,39]. The data were obtained from the source http://www.csbio.sjtu.edu.cn/bioinf/LR_PPI/Data.htm, accessed on 20 May 2020, indicated in [39].

The **Multi-species** data set was proposed by Chen et al. [19] and was obtained by gathering proteins belonging to three organisms—*C. elegans*, *D. melanogaster* and *E.coli*. The data set has been obtained by combining the three protein–protein interactions data sets originally proposed by Guo et al. [40]. In addition to the full combined data set, several non-redundant versions are available, in which proteins with sequence similarity above a certain threshold have been removed. We have used in our experiments the full data set, alongside the data sets https://github.com/muhaochen/seq_ppi, accessed on 20 May 2020, filtered using 25% and 1% similarity thresholds.

An overview of the data sets and their number of positive and negative interactions is presented in Table 1.

The data were preprocessed and features were extracted as described in Section 3.2. The value for the lag parameter (necessary in the computation of AC features) was 30, as suggested by Guo et al. [12,40] for the Multi-species data set, resulting in a number of 14×30=420 features per protein sequence; for the HPRD data set we used a value of 20, resulting in 280 features (this value was chosen considering that the protein with the shortest primary sequence is formed of 25 amino acids).

**Figure 5:**
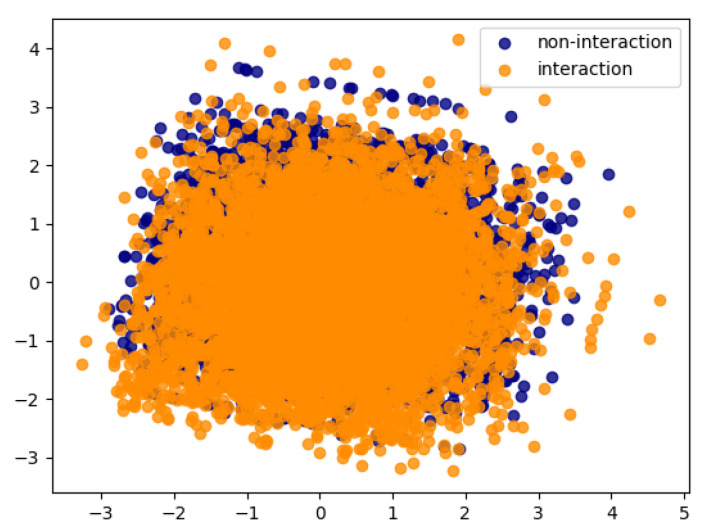
PCA visualization of the data instances for the Multi-species data set.

Figure 5 depicts a two-dimensional PCA projection of the protein pairs for the Multi-species data set, in which pair features are obtained by concatenating the features of the two proteins. The figure shows a low degree of separation between the two classes.

### 4.2. Results

We followed the evaluation protocol used in previous studies [7,19] and performed 10-fold cross-validation for the HPRD data set and 5-fold cross-validation for the multi-species data set. Thus, during each cross-validation step, AutoPPI was trained and validated on the majority of the data sets $C_{+}$ and $C_{-}$ (80% for the Multi-species data sets and 90% for the HPRD data set). After training, AutoPPI is tested on the remaining data (20% for Multi-species and 10% for HPRD) which was not used for training and validation.

Table 2 presents the results for the three architectures proposed in Section 3.3.1 on the data sets described in Section 4.1, following the methodology introduced in Section 3. In the second column of the table the used architecture is depicted: one denotes the Joint–Joint architecture, two corresponds to the Siamese–Joint architecture and three indicates the Siamese–Siamese architecture The values for the performance measures described in Section 3.4.2 were averaged over the cross-validation runs and a 95% CI of the average values is provided. The best performances are highlighted, for each data set.

The results presented in Table 2 reveal very good performances for all three architectures in terms of AUC values raging from 0.96 to 0.985. Generally, the Siamese–Joint and Siamese–Siamese architectures outperform the Joint–Joint variant, thus indicating the importance of capturing common patterns in both proteins involved in the interaction. The best performing model is the Siamese–Siamese architecture (denoted by three in the table) that provided the best AUC values for three out of the four data sets used. Moreover, when inspecting the values for the other evaluation measures we notice that this architecture conveys the best results in most cases (accuracy and $F_{1}$-score: three out of four, precision and specificity: four out of four).

Despite the low degree of separation between the “+” and “−” classes (see Figure 5), AutoPPI succeeded to learn a good decision boundary. The quality of the learned decision surface is reflected in high values obtained for the performance metrics. As expected, the autoencoders $AE_{+}$ and $AE_{-}$ were able to learn encodings (through their latent representations) able to distinguish between the class of proteins that interact and the class of proteins that do not interact.

With regard to the data sets, we observe that our models have good performances, irrespective of data set size and degree of imbalance. This is best noticed when inspecting the three versions of the Multi-species data set, where the AUC measure is above 0.98 for all three, with very small differences between obtained values, with respect to all evaluation measures.

Last, but not least, we note very small values obtained for the CI (below 0.01) expressing the stability of our model.

## 5. Comparison to Related Work

Table 3 presents a comparison between our best model on the HPRD data set, AutoPPI with Siamese–Joint architecture, and various machine learning and deep learning approaches that use protein sequence data, tested on the same data set. The machine learning and deep learning methods include support vector machines (SVMs) trained on CT and AC features or other representations derived from the protein sequence [20,40,41], parallel SVMs [42], SVMs used in combination with a compressed sensing algorithm for reducing the input space dimensionality (CS-SVM) [43], extreme learning machines (ELM) [44], a Latent Dirichlet allocation model combined with a Random Forest (LDA-RF) [39], a stacked autoencoder [7], a graph variational autoencoder [25], deep neural networks [45,46,47] and a deep convolutional recurrent neural network [19]. From the results presented in the table, we can see that AutoPPI outperforms classical machine learning algorithms, with the exception of the LDA-RF approach proposed by Pan et al. [39], is slightly surpassed by the S-VGAE proposed in [25] and achieves comparable performance with the stacked autoencoder proposed by Sun et al. [7] (marginally outperforming it). We note that the LDA-RF, ELM, CS-SVM and S-VGAE methods performed a 5-fold cross-validation evaluation procedure, while the other studies employed a 10-fold methodology.

Table 4 presents a comparison between our best model on the Multi-species data set, AutoPPI with Siamese–Siamese architecture, and the deep residual recurrent convolutional architecture proposed by Chen et al. [19].

The comparative results illustrated in Table 3 highlight that our AutoPPI approach outperforms the related work on the HPRD data set, except for three cases: the S-VGAE approach, which is a graph-based method that exploits network information in PPIs and two deep neural networks (the PIPR method, which is a deep neural network that models long range dependencies and patterns in protein sequences through bidirectional gated recurrent units networks, and DNN-CTAC). On all Multi-species data sets, our approach has higher performance than the related approaches [19], in terms of *accuracy* and F1-score. Overall, in 81.25% of the comparisons with the related work (in 13 cases out of 16 comparisons), AutoPPI provides better results.

For verifying the statistical significance of the performance improvement brought by AutoPPI compared to the approaches considered in Table 3 and Table 4, a one tailed paired Wilcoxon signed-rank test [48,49] was applied. The sample of values obtained for all evaluations and both *accuracy* and *F1* performance metrics described in Table 3 and Table 4 for AutoPPI was tested against the sample of values obtained for the related work. A *p*-value of 0.00094 was obtained, showing that the performance improvement of our method with respect to the related work approaches is statistically significant, at a significance level of alpha=0.01.

## 6. Conclusions

Living beings are complex machinery whose inner gears have evolved to work flawlessly, in ideal conditions. Proteins, the fundamental cogwheels in this gear, perform most of their functions in complexes and thus understanding the way they interact with each other as well as with other molecules represents a step forward in figuring out the mechanisms of life. Through the present work we aim to bring our contribution in this endeavour and to advance the state of in silico methods for solving the problem of PPI prediction.

We propose a procedure for the binary classification of protein–protein interactions (positive versus negative) having as focal points two autoencoders that are trained to encode relationships between proteins that do or do not interact. In addition to protein interactions, the input data are represented by protein primary sequences, which are encoded by a combination of two types of descriptors: conjoint triad and autocovarriance. We propose three types of architectures for the autoencoders and evaluate our approach on four data sets including proteins from different species. Experimental results demonstrate the potential of our approach, which proves to be highly competitive in relation to other solutions proposed in the literature. To the best of our knowledge autoencoders have not been exclusively used for predicting PPI so far (in all other approaches autoencoders provide an intermediate assistant used for feature extraction and dimensionality reduction in data before this is fed to the main classifier).

We note the generality of the binary classification model AutoPPI introduced in this paper. The current approach used features extracted from pairs of protein sequences, but sequence alignments and position-specific scoring matrices may be used as input data for AutoPPI as well. Even if it has been applied and evaluated for *protein–protein interaction*, it is general and may be applied for other binary classification problems.

To further evaluate the performance of AutoPPI, its experimental evaluation will be extended on other data sets from the PPI literature, including PPI networks. Given the fact that experimental methods of obtaining PPIs are expensive and prone to generating false positives, PPIs databases are being continuously updated. In order to more reliably asses the performance of our approach from this perspective, we plan to further evaluate it on more recently curated PPI data sets. In addition, alternative representations for the proteins will be envisaged, such as matrices derived from protein sequences, evolutionary information and word embeddings representations for these sequences. In this context, we plan to investigate deep architectures, formed of convolutional and recurrent layers, which could skip the additional step of representing proteins using fixed-length representations and better extract sequential information involved in protein interactions. For assessing the generality of the AutoPPI model, it will be applied and evaluated on sequence alignments instead of protein sequences as well as on other classification problems, such as the identification of protein–RNA interactions, the prediction of protein–protein interaction sites or protein family classification.

## Figures and Tables

**Table 1:** Data sets used in the experiments.

Data Set	Number of Positive Interactions	Number of Negative Interactions
HPRD	36,630	36,480
Multi-species	32,959	32,959
Multi-species < 0.25	19,458	15,827
Multi-species < 0.01	10,747	8065

**Table 2:** Experimental results. 95% CIs are used for the results. 1—denotes the Joint–Joint architecture, 2—the Siamese–Joint architecture, 3—the Siamese–Siamese architecture. The best performances are marked in bold.

Data Set	Arch.	Accuracy	$F_{1}-\mathrm{Score}$	Precision	Recall	Specificity	AUC
**HPRD**	1	0.977 ± 0.0006	0.977 ± 0.0007	0.986 ± 0.0009	0.968 ± 0.001	0.986 ± 0.0009	0.977 ± 0.0006
	2	**0.979 ± 0.0007**	**0.979 ± 0.0007**	0.973 ± 0.0015	**0.985 ± 0.009**	0.973 ± 0.0015	**0.979 ± 0.0007**
	3	0.96 ± 0.0014	0.959 ± 0.0015	**0.992 ± 0.006**	0.928 ± 0.0024	**0.992 ± 0.006**	0.960 ± 0.0014
**Multi-species**	1	0.97 ± 0.0007	0.969 ± 0.0006	0.995 ± 0.0007	0.944 ± 0.0015	0.995 ± 0.0006	0.97 ± 0.0005
	2	0.969 ± 0.0008	0.97 ± 0.0009	0.965 ± 0.0028	**0.974 ± 0.002**	0.964 ± 0.0025	0.97 ± 0.008
	3	**0.982 ± 0.0008**	**0.982 ± 0.0008**	**1 ± 0**	0.964 ± 0.0016	**1 ± 0**	**0.982 ± 0.008**
**Multi-species** **<0.25**	1	0.973 ± 0.0011	0.975 ± 0.0009	0.995 ± 0.0011	0.956 ± 0.0017	0.995 ± 0.0012	0.975 ± 0.001
	2	0.976 ± 0.0007	0.978 ± 0.0008	0.974 ± 0.0011	**0.983 ± 0.0008**	0.968 ± 0.0013	0.975 ± 0.0008
	3	**0.983 ± 0.0015**	**0.984 ± 0.0014**	**1 ± 0**	0.969 ± 0.0027	**1 ± 0**	**0.985 ± 0.0013**
**Multi-species** **<0.01**	1	0.972 ± 0.0023	0.975 ± 0.0019	0.993 ± 0.001	0.958 ± 0.0035	0.991 ± 0.0015	0.975 ± 0.002
	2	0.978 ± 0.0015	0.981 ± 0.0013	0.975 ± 0.0024	**0.987 ± 0.0027**	0.966 ± 0.0031	0.976 ± 0.0015
	3	**0.981 ± 0.0016**	**0.983 ± 0.0014**	**1 ± 0**	0.966 ± 0.0027	**1 ± 0**	**0.983 ± 0.0014**

**Table 3:** Comparison between our method and related work on the HPRD data set.

Method	Accuracy	F1
**AutoPPI**	0.979 ± 0.0007	0.979 ± 0.0007
SAE [7]	0.9719	-
PIPR [19]	0.9811	0.9803
LDA-RF [39]	0.979 ± 0.005	-
CT-SVM [20] reported in [7]	0.83	-
AC-SVM [40] reported in [7]	0.9037	-
Parallel SVM [42] reported in [7]	0.9200–0.9740	-
ELM [44] reported in [7]	0.8480	0.8477
CS-SVM [43]	0.941	0.937
SVM [41]	0.942	-
DNN [46]	0.9443 ± 0.0036	-
DNN-PPI [45]	0.9726 ± 0.0018	-
DNN-CTAC [47]	0.9837	-
S-VGAE [25]	0.9915 ± 0.0011	0.9915 ± 0.0012

**Table 4:** Comparison between our method and related work on the Multi-species data sets.

Data Set	Method	Accuracy	F1
Multi-species	AutoPPI	0.9821± 0.0008	0.9818 ± 0.0008
	PIPR [19]	0.9819	0.9817
Multi-species <0.25	AutoPPI	0.9829 ± 0.0015	0.9842 ± 0.0014
	PIPR [19]	0.9791	0.9808
Multi-species <0.01	AutoPPI	0.9808 ± 0.0016	0.9829 ± 0.0014
	PIPR [19]	0.9751	0.9780

## Data Availability

The data sets used in the study are publicly available and can be accessed using the following links: http://www.csbio.sjtu.edu.cn/bioinf/LR_PPI/Data.htm, accessed on 20 May 2021. for the HPRD dataset and https://github.com/muhaochen/seq_ppi, accessed on 20 May 2021 for the Multi-species dataset.
